# A Non-Linear Osteometric Modeling Method for Three-Dimensional Mandibular Morphological Changes During Growth: One-Year Monitoring of Miniature Pigs Using Cone-Beam Computed Tomography

**DOI:** 10.3389/fbioe.2022.854880

**Published:** 2022-05-24

**Authors:** Hsien-Shu Lin, Tung-Wu Lu, Jia-Da Li, Pei-An Lee, Yunn-Jy Chen

**Affiliations:** ^1^ School of Dentistry, National Taiwan University, Taipei, Taiwan; ^2^ Department of Biomedical Engineering, National Taiwan University, Taipei, Taiwan; ^3^ Department of Orthopaedic Surgery, School of Medicine, National Taiwan University, Taipei, Taiwan; ^4^ Department of Dentistry, National Taiwan University Hospital, Taiwan, China

**Keywords:** maxillofacial development, swine, miniature, 3D morphological change, osteometric scaling

## Abstract

Knowledge of mandibular growth and development is essential for diagnosis of malformation and early interception. A previous method of quantifying mandibular growth using the distances between selected anatomical landmarks over the growth period does not provide a complete, quantitative description of the continuous growth patterns. The current study aimed to bridge the gap by measuring the 3D continuous growth of the mandible in miniature pigs using cone-beam computerized tomography (CBCT). The mandibles of the pigs were CBCT-scanned monthly over 12 months, and the 3D mandibular models were reconstructed. A new non-linear, time-dependent osteometric modeling approach was developed to register two consecutive mandible models by searching for the corresponding points with the highest likelihood of matching the anatomical and morphological features so that the morphological changes patterns for each month could be described using color maps on the models. The morphological changes of the mandible were found to decrease anteriorly, with the condyle region and the posterior part of the ramus growing faster than the rest of the mandible. The condyle region showed the fastest growth rate and the posterior ramus the second during the growth period, while the middle and anterior corpus regions showed the slowest growth rates. In conclusion, the current results revealed the non-linear patterns and rates of morphological changes in different growth regions and the whole mandible. The new approach may also be useful for future studies on the growth of the mandible in other animals.

## Introduction

The mandible is the strongest, largest and lowest bone in the human face (Nirmale et al., 2012), affecting orofacial functions, occlusion, and jaw relationships (Drevenšek et al., 2005). Knowledge of mandibular growth and development is essential for malformation diagnosis and early interception (So, 1997; Efstratiadis et al., 1999). Data on monthly morphological changes during mandibular growth will provide useful information for the establishment of such knowledge. However, bone growth is a very complex process during which the shape and structure of the bone are modified *via* a dynamic process involving both bone resorption and deposition (Enlow and Harris, 1964; Gu and McNamara Jr, 2007), indicating that mandibular growth may not be a simple homogeneous (uniform) expansion of the bone (Pal, 2014; Kelly et al., 2017). Therefore, techniques for accurate modeling and analysis of regular 3D imaging data over the growth period are essential for the quantitative description of the non-uniform morphological changes of the mandible.

Cephalograms are often used as the basis for diagnostic imaging and have been used to image mandible shape changes at limited time instances in a limited number of *in vivo* studies (Nanda, 1955; Liu et al., 2010). Bjork (1963) was the first to use overlapping lateral cephalometric radiographs to observe the growth of the mandible implanted with tantalum metal markers in children *in vivo* (Björk, 1963). However, using cephalograms for long-term, *in vivo* measurement of human mandible growth is not feasible for ethical reasons because it requires multiple exposures to ionizing radiation over the monitoring period. On the other hand, the use of cephalograms in monitoring the growth of the maxillofacial regions of miniature pigs has been well documented because they are similar to humans in the morphology of the mandible (size and shape) and their bone metabolism rate (Ström et al., 1986; Langenbach et al., 2002; Huh et al., 2006) (Ide et al., 2013; Holton et al., 2015). More recent development in computed tomography (CT) enables accurate three-dimensional (3D) reconstruction of the bone and the measurement of its morphology (Adams et al., 2004; Cavalcanti et al., 2004) (Broadbent, 1981; Mah and Hatcher, 2003; Adams et al., 2004). Three-dimensional CT has been used to study human mandibular growth using cadaveric specimens of different growth stages (Kelly et al., 2017; Schipper et al., 2021), or in patients with diseases that require follow-up with CT imaging (Cavalcanti et al., 2004; Carvalho et al., 2010). However, the adverse effects of ionizing radiation on living bodies preclude the long-term monitoring using CT *in vivo*. While low-dose, dental Cone-Beam-Computed-Tomography (CBCT) allowed relatively straightforward and rapid to obtain 3-D images of the mandible (Hildebolt et al., 1990; Cavalcanti and Vannier, 1998; DeCoster et al., 2012), the radiation dose is still not low enough for long-term monitoring of the human mandibular growth. Therefore, miniature pigs remain a good alternative to humans in mandibular growth studies.

Measurement of the mandibular growth in miniature pigs using CBCT has been reported by Lin H.-S. et al. (2014). The development of the mandible was quantified by measuring the magnitudes and average rates of monthly changes of the three-dimensional distances between selected anatomical landmarks over the growth period. While this approach revealed the length changes of a limited number of bone segments of the mandible in a selection of positions and directions during growth, it does not provide the quantitative, continuous patterns of morphological changes of the mandibular bone during growth. Methods are needed for a complete description of the continuous changes in the 3D morphology of the mandible from measurements at a series of discrete-time instants during growth.

The purpose of the current study was to develop a new approach based on 3D time-dependent, non-linear osteometric modeling of the bones to describe the 3D quantitative, continuous patterns of the morphological changes of the mandible during growth from the CBCT data of miniature pigs taken monthly from the age of one month onwards over 12 months.

## Material and Methods

### Subjects

Eight Lee-Sung strain miniature pigs (4 males and 4 females) raised on a certified farm for experimental animals (temperature: 26–28°C; humidity: 55%–60%) were used in the current study. Lee-Sung strain miniature pigs are often used in animal studies in Asia (Moran et al., 2016; Ju et al., 2019). From one month onwards, each of the pigs was given a CBCT scan of the mandible once every four weeks over a year. To avoid differences in the number of days between calendar months and to simplify the description of the changes over the time intervals, a time interval of four weeks was referred to as “a month” (T = time interval = 4 weeks), so a total of twelve sets of CBCT data were obtained for each pig (T = T1, …, T12). According to Cheng et al. (2010), the milk dentition period of the miniature pigs occurs during the first 18 weeks, equivalent to 0.5–5 years for tooth eruption in humans; and the mixed dentition period of the pigs at 18–64 weeks was equivalent to 6–12-year old human growth. The current study measured 12 instances over the early growth of 48 weeks, corresponding to nearly 2/3 of the mixed dentition stage. The pigs were under general anesthesia during each CT scan by an intramuscular injection of 1 cc/10 kg of zoletil 50 (50 mg/kg) (Virbac Laboratories, Carros, France). To prevent choking, saliva production was inhibited by an intramuscular injection of atropine sulfate (Antopin, 1 mg/ml; 0.5 cc: < 20 kg; 1 cc: >20 kg; Sinton Chem & Pharm Co. Ltd., Taiwan). The CT scanning was performed using a low radiation dose CBCT system (i-CAT, Imaging Sciences International, Inc., United States) operating at a tube potential of 120 kVp and a tube current of 3–8 mA, giving images with a voxel size of 0.4 mm × 0.4 mm × 0.4 mm and a greyscale intensity of 12 bits. The field of view was 22 cm (height) × 16 cm (diameter) with the Extended Field of View model provided by the system. During the CBCT scan, the pig was restrained on a purpose-built workbench using transparent tapes. The mandible was positioned within the center of the region of interest with the guidance of an optical localizer in the shape of a cross. This study was carried out according to the recommendations in the *Guide for the Care and Use of Laboratory Animals* by the National Institutes of Health (Lin H.-S. et al., 2014). The protocol was approved by the Committee on the Ethics of Animal Experiments of the National Taiwan University (Permit Number: 20080124).

### Three-Dimensional Non-linear Osteometric Modeling of the Mandible

To quantify the magnitudes and rates of morphological changes of the mandible during growth over the duration of the monitoring, 3D models of the mandible were reconstructed from the CT data sets for each subject at each monitoring instance using a commercial image-processing package (Amira, Visage Imaging Inc., United States). A new time-dependent, non-linear osteometric modeling approach was developed to describe the growth over time.

The new osteometric modeling approach was based on the registration and time-fitting of the 3D model of a subject at each of the instances to the model at the subsequent instance, i.e., the model at the *i*th instance (T_*i*_) to the model at the (*i*+1)^th^ instance (T_*i*+1_), starting from the first month (*i* = 1). Given the 3D model of the mandible described by *n* vertices at the current (*i*th) instance, the purpose of the registration was to find the corresponding vertices on the model at the (*i*+1)^th^ instance. For the *j*th vertex on the *i*th model, the search for the corresponding vertex on the (*i*+1)^th^ model was to find the rotation matrix (R_*i*_) and a translation vector (**v**_*i*_) of the *i*th model relative to the (*i*+1)^th^ model to maximize the correlation between the neighboring vertices of the *j*th vertex on the *i*th model and a set of neighboring vertices on the (*i*+1)^th^ model. The center vertex of the set of neighboring vertices on the (*i*+1)^th^ model was then marked as the *j*th vertex on the (*i*+1)^th^ model. The above optimization problem could be described mathematically as follows.

Given the position vector of the *j*th vertex on the *i*th model (**P**_*i,j*_) and the position vectors of the vertices within the neighborhood of a radius of *r* (**Q**_*i,j*_), the problem associated with identifying the corresponding *j*th vertex on the (*i*+1)^th^ model was to find the rotation matrix (R_*i*_) and translation vector (**v**_*i*_) of the *i*th model relative to the (*i*+1)^th^ model to maximize the correlation coefficient between **Q**_*i,j*_ on the *i*th model and **Q**_***(*** *i+1),j*_ on the (*i*+1)^th^ model.

$$\max.f=\text{corr}\left({\text{Q}}_{\text{i},\text{j}},{\text{Q}}_{\left(\text{i}+1\right),\text{j}}\right)=\frac{cov\left(Q_{\text{i},j},Q_{\left(\text{i}+1\right),\text{j}}\right)}{\sigma_{i,j}\sigma_{\left(i+1\right),j}}$$ (1)

subject to

$${\text{P}}_{\left(\text{i}+1\right),\text{j}}={\text{R}}_{\text{i}}{\text{P}}_{\text{i},\text{j}}+{\text{v}}_{\text{i}}$$ (2)

where σ_*i,j*_, σ_*(i+1),j*_ and *cov* were standard deviations of and covariance between Q_*i,j*_ and Q_*(i+1),j*,_ respectively. The above optimization problem was solved for each of the vertices on the *i*th model to identify the corresponding vertices on the (*i*+1)^th^ model.

### Calculation of Rates of Morphological Changes in Minipigs

**FIGURE 1:**
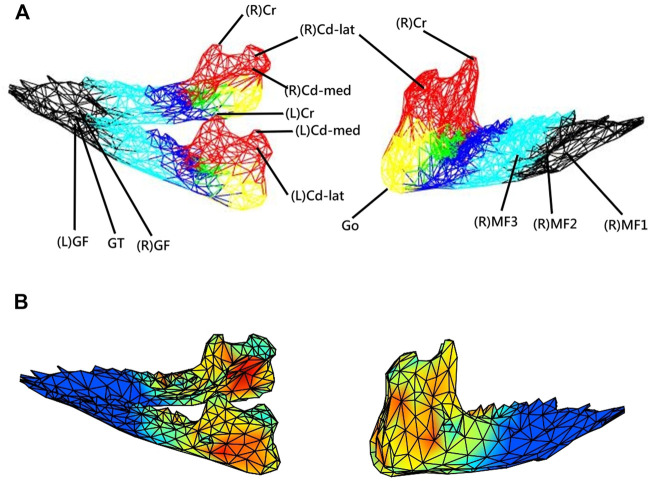
**(A)** Three-dimensional (3D) model of the mandible indicating 17 anatomical landmarks and six anatomical parts (regions) selected to indicate the distribution of the morphological changes of the mandible during growth, namely condyle (red), posterior ramus (yellow), anterior ramus (green), posterior corpus (dark blue), middle corpus (light blue), and anterior corpus (black). **(B)** Each mandible at each measurement instance was reconstructed from CBCT data and described by a model of a 3D mesh of vertices forming about 653 triangular elements. With the subject-specific model, the rates of morphological changes (growth rates) were shown with different colors as a color map on the model.

Once the correspondence of vertices was established between the *i*th and (*i*+1)^th^ models, the increase of the distance between any two adjacent vertices, *j*th and (*j*+1)^th^ vertices, from T_*i*_ to T_*i+1*_ was defined as the monthly morphological change (growth) of the vertex pair at T_*i+1*_. These monthly morphological changes were calculated and normalized by the lengths of the vertices at T_*i*_ over the monitoring period. For calculating the rate of morphological changes of a pair of vertices, a cubic spline was fitted to the normalized monthly morphological changes over the period of monitoring (12 months in the current study) in a least-squares sense. The gradient of the curve at T_*i*_ then gave the rate of morphological change of the vertex pair at that instance. The rate of morphological change of each vertex was calculated as the average value of all vertex pairs defined by the current vertex (Figure 1).

The new approach had the capability of 3D volumetric modeling and analysis of the morphological data obtained from cone-beam CT, enabling further feature extractions, including data along any given direction or component. In the current study, as a first application, the new approach was used to measure and describe the 3D surface morphological changes during growth in mini pigs. For this purpose, each mandible was described by a 3D mesh of 653 vertices with 1326 triangular elements on the surface of the mandible (Figure 1). The total number of vertices chosen was determined via sensitivity analysis. The mandible was first modeled with more than 6500 vertices (data points), and then the vertices were reduced sequentially at a decrement of 5% of the total number of vertices until the per cent morphological changes showed a significant difference. The second last number of vertices, i.e., 653 vertices, was then chosen for the current study.

### Validation of the New Approach

For the assessment of the accuracy of the new approach, 17 anatomical landmarks (validation points) on each of the CBCT-based 3D mandibular models of all eight subjects were identified using a previously established method, which is highly reliable in determining the selected anatomical landmarks (Lin H.-S. et al., 2014; Lin et al., 2014b) (Table 1 and Figure 1). The anatomical landmarks Cd-lat, Cr, and Go were identified automatically using their geometrical features following a geometric approach used by Lin et al. (2015) and further verified by an experienced dentist (HSL). The other anatomical landmarks were identified manually by the same dentist (HSL) within Geomagic 3D Software (Geomagic, Inc., United States). The reliability of this procedure was determined by the same dentist repeatedly identifying the landmarks, giving an Intra-Class Correlation Coefficient (ICC) of 0.9 (Lin H.-S. et al., 2014; Lin HS. et al., 2014). The position accuracy of the current method was then described by the root mean squared errors (RMSE) of position measurements against the gold standard positions determined using the previously established method (Lin H.-S. et al., 2014). On the other hand, the accuracy in measuring the rates of morphological changes was assessed by the RMSE of the model-calculated rates of changes of the inter-landmark distances against the gold standard. The rates of changes were calculated as the inter-landmark distance differences between T_*i*_ and T_*i+1*_ divided by the inter-landmark distance at T_*i*_.

**TABLE 1:** A total of 17 anatomical landmarks on the mandible were used as validation points to monitor the 3D morphological changes of the mandibular surface during growth.

Landmark	Symbol	Definition
lateral pole of condyle	Cd-lat	The most protruding point on the lateral side of the mandibular condyle
medial pole of condyle	Cd-med	The most protruding point on the medial side of the mandibular condyle
coronoid process	Cr	The most protruding point on the coronoid
Gonion	Go	The most posterior and inferior point at the mandibular angle
anterior mental foramen	MF1	The most anterior edge of the export of the mental nerve
middle mental foramen	MF2	The middle edge of the export of the mental nerve
posterior mental foramen	MF3	The most posterior leading edge export of the mental nerve
genial fovea	GF	The small fovea on both sides of the genial tubercle
genial tubercle	GT	The most prominent point of the genial tubercle

### Description of the Rates of Morphological Changes (Growth Rates) During Growth

In the current study, for simplicity, the term “growth rate” was used interchangeably with the “rate of morphological changes” to indicate the outcome of bone modeling and remodeling. It is acknowledged that the rate of morphological changes does not reveal the bone resorption and deposition within the mandible. The calculated rates of morphological changes for each of the vertices on the mandible model at any instance were indicated at the vertex using different colors. Therefore, the growth patterns of the whole mandible could be described using color maps on the models at the instances over twelve months. The color map could be visualized in three dimensions via the GUI of a house-developed software system using MATLAB (MathWorks, Inc., United States). For the comparison of the observed growth patterns in the current study with those in the literature, the mandible was divided into six anatomical regions, namely condyle, posterior ramus, anterior ramus, posterior corpus, middle corpus, and anterior corpus, and the average rates of morphological changes (growth rates) across all the vertices for each region were obtained (Figure 1). There were 171, 49, 42, 131, 130, and 130 vertices in the six regions, respectively. Means and standard deviations (SD) of the monthly average rates of anterior/posterior, superior/inferior, and medial/lateral morphological changes (growth rates) over each of the six regions of the mandible over the monitoring period of one year were obtained. The changes in the mean thickness of three selected anatomical regions, i.e., anterior and posterior ramus regions and the mediolateral width of the condylar head, were also calculated from T1 to T12.

## Results

The RMSE of the positions between the new method and the gold standard for the positions of the 17 anatomical landmarks over the 12 months of monitoring were all less than 0.86 mm (Table 2). The RMSE were all less than 0.44 mm over the first eight months of monitoring and 0.86 mm over months 9–12 (Table 2). The average of the RMSE of 17 anatomical landmarks ranged between 0.17 and 0.66. The average of the RMSE of 17 anatomical landmarks was less than 0.29 over the first eight months of monitoring and 0.66 mm over months 9–12 (Table 2). The RMSE of the rates of inter-landmark distance changes between the new method and the gold standard over the 12 months of monitoring were mostly less than 0.05%/month (Table 3). The average of the RMSE of the rates of inter-landmark distance changes was less than 0.06%/month over the first eight months of monitoring and 0.08%/month over months 9–12 (Table 3).

**TABLE 2:** Root mean square errors (RMSE) of the positions between the new method and the gold standard for the 17 anatomical landmarks (validation points) across all subjects at 12 instances over the 48-week monitoring period. (*n* = 8; unit: mm).

Landmark	Measurement instance													
	T1	T2	T3	T4	T5	T6	T7	T8	T9	T10	T11	T12	Mean	SD
GT	0.22	0.38	0.03	0.37	0.02	0.21	0.05	0.21	0.54	0.68	0.54	0.68	0.33	0.24
Right side														
Cd-lat	0.25	0.15	0.34	0.23	0.1	0.37	0.25	0.25	0.51	0.51	0.48	0.83	0.36	0.20
Cd-med	0.20	0.13	0.4	0.41	0.23	0.27	0.12	0.15	0.36	0.52	0.64	0.77	0.35	0.21
Cr	0.29	0.23	0.24	0.24	0.14	0.27	0.2	0.18	0.41	0.54	0.54	0.66	0.33	0.17
MF1	0.24	0.31	0.18	0.31	0.12	0.23	0.11	0.24	0.71	0.86	0.72	0.66	0.39	0.27
MF2	0.22	0.26	0.11	0.32	0.12	0.22	0.09	0.18	0.62	0.77	0.67	0.75	0.36	0.26
MF3	0.10	0.28	0.07	0.25	0.09	0.24	0.18	0.16	0.62	0.71	0.61	0.65	0.33	0.24
GF	0.15	0.26	0.08	0.43	0.06	0.26	0.07	0.3	0.62	0.75	0.62	0.62	0.35	0.25
Go	0.24	0.21	0.31	0.24	0.15	0.30	0.25	0.33	0.34	0.43	0.49	0.50	0.32	0.11
Left side														
Cd-lat	0.26	0.21	0.43	0.12	0.33	0.37	0.22	0.21	0.41	0.55	0.41	0.55	0.34	0.14
Cd-med	0.20	0.11	0.29	0.34	0.22	0.22	0.12	0.24	0.31	0.38	0.56	0.70	0.31	0.17
Cr	0.19	0.22	0.2	0.17	0.32	0.23	0.09	0.25	0.39	0.46	0.52	0.68	0.31	0.17
MF1	0.18	0.36	0.28	0.26	0.12	0.40	0.25	0.16	0.73	0.77	0.56	0.47	0.38	0.22
MF2	0.16	0.25	0.10	0.23	0.1	0.3	0.17	0.21	0.69	0.85	0.79	0.80	0.39	0.30
MF3	0.19	0.27	0.22	0.24	0.22	0.34	0.25	0.26	0.53	0.52	0.52	0.53	0.34	0.14
GF	0.14	0.24	0.06	0.38	0.15	0.34	0.08	0.32	0.58	0.65	0.53	0.57	0.34	0.21
Go	0.27	0.19	0.19	0.28	0.44	0.37	0.31	0.38	0.38	0.37	0.46	0.75	0.37	0.15
Mean	0.21	0.24	0.21	0.28	0.17	0.29	0.17	0.24	0.51	0.61	0.57	0.66		
SD	0.05	0.07	0.12	0.08	0.11	0.06	0.08	0.07	0.14	0.16	0.10	0.11		

**TABLE 3:** Root mean square errors (RMSE) of the monthly growth rates between the new method and the gold standard across all subjects for landmark pairs calculated as the inter-marker length changes between two consecutive instances normalized to the corresponding length at the proceeding instance over the monitoring period. (*n* = 8; unit: %/month).

	Measurement interval												
	T1-T2	T2-T3	T3-T4	T4-T5	T5-T6	T6-T7	T7-T8	T8-T9	T9-T10	T10-T11	T11-T12	Mean	SD
MF1-Go	0.04	0.03	0.05	0.04	0.04	0.03	0.04	0.05	0.10	0.02	0.03	0.04	0.02
Go-MF3	0.04	0.05	0.07	0.05	0.05	0.04	0.08	0.03	0.10	0.05	0.03	0.05	0.02
Cr-Cd-lat	0.10	0.03	0.09	0.05	0.02	0.10	0.06	0.09	0.07	0.10	0.05	0.07	0.03
Cr-Cd-med	0.04	0.02	0.01	0.07	0.03	0.08	0.02	0.09	0.09	0.08	0.08	0.05	0.03
MF1-MF2	0.10	0.03	0.05	0.02	0.00	0.06	0.02	0.02	0.04	0.03	0.01	0.04	0.03
MF2-MF3	0.04	0.03	0.00	0.05	0.06	0.03	0.01	0.04	0.03	0.03	0.01	0.04	0.02
Go-Cr	0.03	0.03	0.04	0.06	0.02	0.03	0.02	0.09	0.08	0.06	0.11	0.05	0.03
Go-Cd-med	0.03	0.04	0.06	0.06	0.03	0.04	0.03	0.14	0.07	0.08	0.13	0.06	0.04
Go-Cd-lat	0.02	0.05	0.04	0.09	0.04	0.02	0.03	0.11	0.09	0.09	0.11	0.06	0.03
MF1-Cd-lat	0.03	0.04	0.04	0.03	0.04	0.02	0.02	0.07	0.06	0.04	0.03	0.04	0.02
MF1-Cd-med	0.02	0.03	0.02	0.02	0.03	0.01	0.01	0.08	0.05	0.06	0.01	0.03	0.02
MF1-Cr	0.03	0.04	0.02	0.02	0.03	0.01	0.02	0.06	0.04	0.03	0.00	0.03	0.02
GF-Cd-lat	0.04	0.02	0.03	0.04	0.03	0.01	0.02	0.08	0.05	0.03	0.02	0.03	0.02
GF-Cd-med	0.02	0.04	0.04	0.01	0.03	0.01	0.02	0.08	0.01	0.07	0.03	0.03	0.02
GF-Cr	0.02	0.04	0.02	0.02	0.03	0.01	0.03	0.07	0.02	0.05	0.03	0.03	0.02
inter-Go	0.04	0.00	0.01	0.04	0.01	0.04	0.02	0.08	0.15	0.03	0.01	0.04	0.04
inter-Cd-lat	0.07	0.05	0.10	0.12	0.12	0.08	0.11	0.09	0.15	0.18	0.07	0.10	0.04
inter-Cr	0.13	0.02	0.04	0.04	0.03	0.04	0.07	0.08	0.03	0.06	0.16	0.06	0.04
inter-Cd-med	0.15	0.03	0.04	0.05	0.02	0.01	0.04	0.09	0.08	0.05	0.20	0.07	0.06
inter-MF3	0.14	0.04	0.08	0.01	0.01	0.02	0.06	0.10	0.10	0.13	0.08	0.07	0.05
inter-MF2	0.09	0.03	0.13	0.05	0.03	0.01	0.08	0.14	0.09	0.07	0.07	0.07	0.04
inter-MF1	0.14	0.00	0.13	0.04	0.02	0.06	0.10	0.13	0.03	0.10	0.08	0.08	0.05
Mean	0.06	0.03	0.05	0.05	0.03	0.03	0.04	0.08	0.07	0.07	0.07		
SD	0.04	0.01	0.04	0.02	0.02	0.03	0.03	0.03	0.04	0.04	0.05		

**FIGURE 2:**
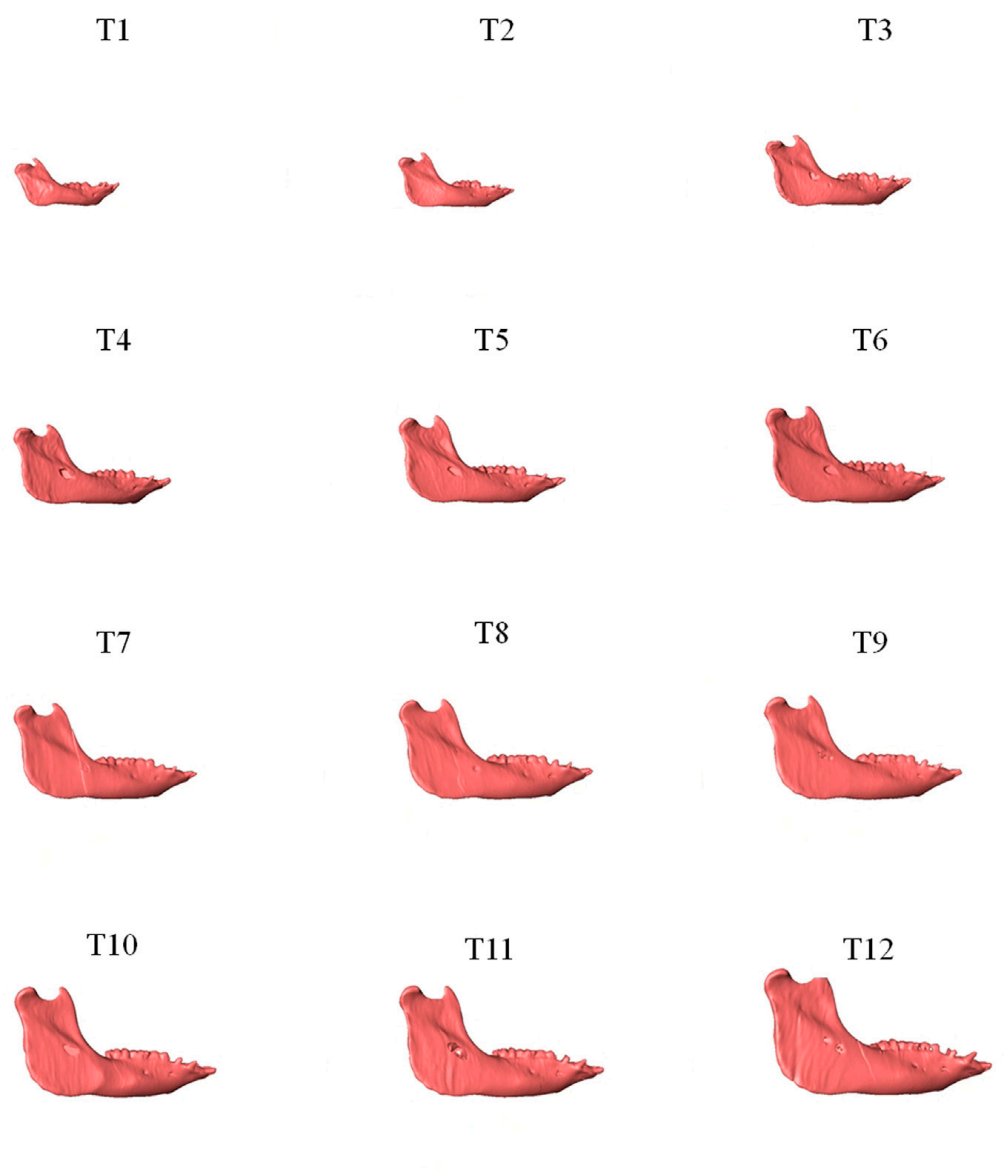
Lateral view of the mandibles of a typical subject showing monthly size and shape changes over the growing period from T1 to T12.

**FIGURE 3:**
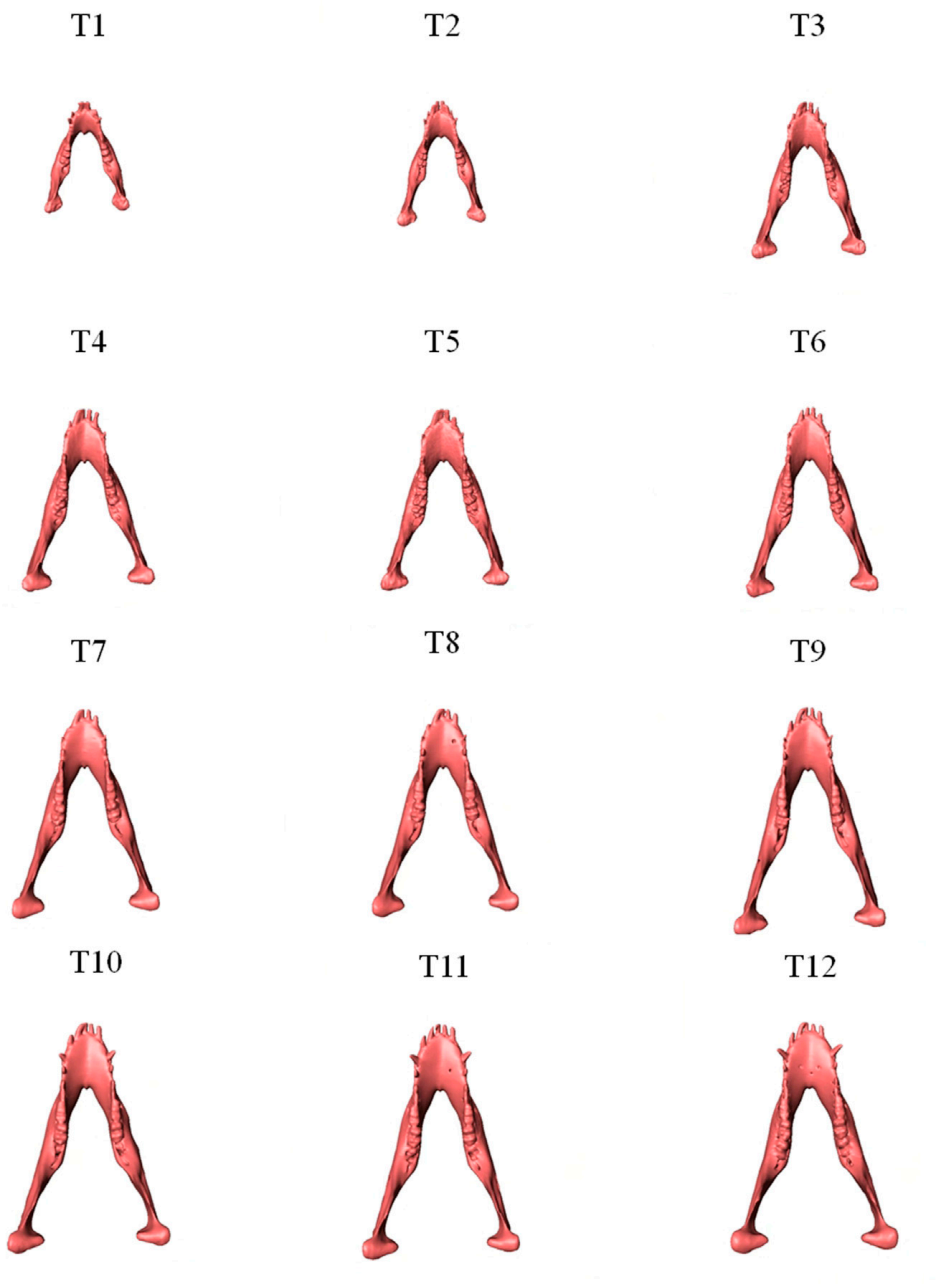
Superior view of the mandibles of a typical subject showing monthly size and shape changes over the growing period from T1 to T12.

**FIGURE 4:**
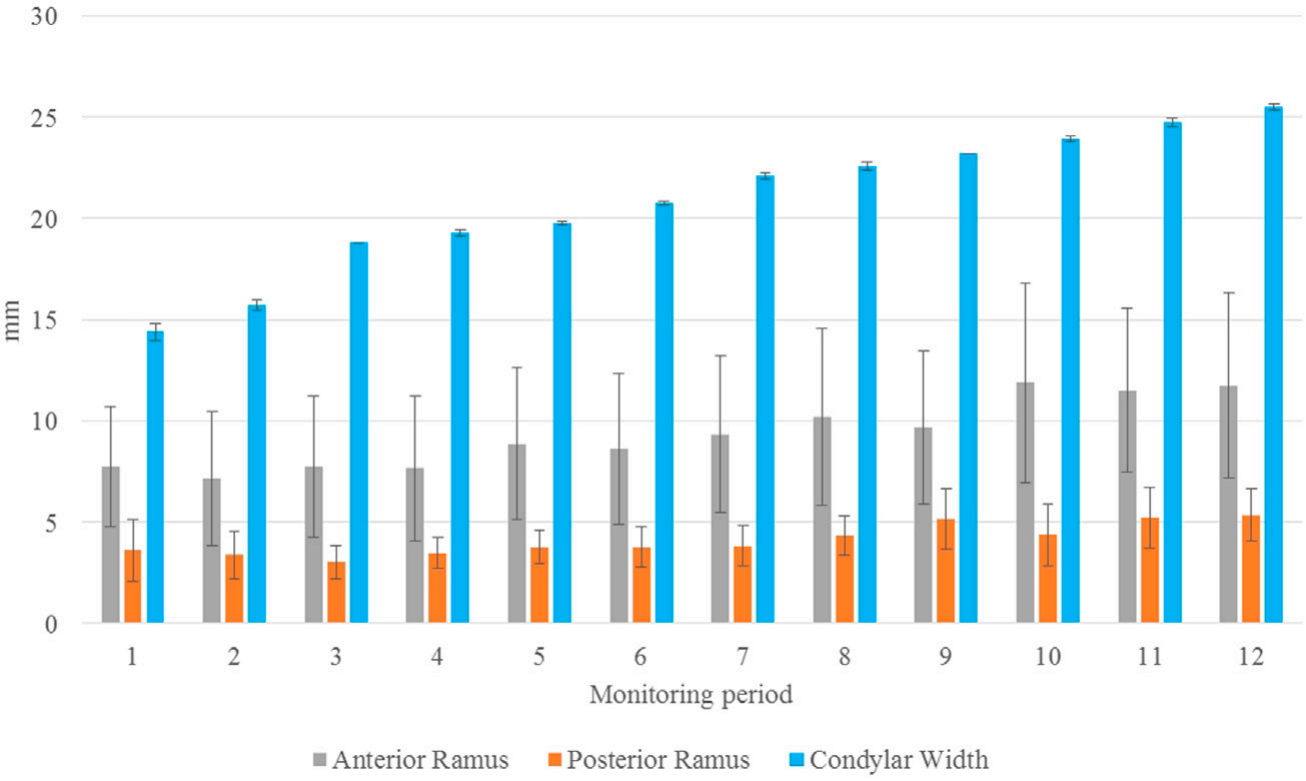
Mean thickness of the anterior (gray) and posterior (orange) ramus regions and the mediolateral length of the condylar head (blue) over the monitoring period of one year from T1 to T12. Standard deviations are indicated as error bars. (*n* = 8; unit: mm).

**FIGURE 5:**
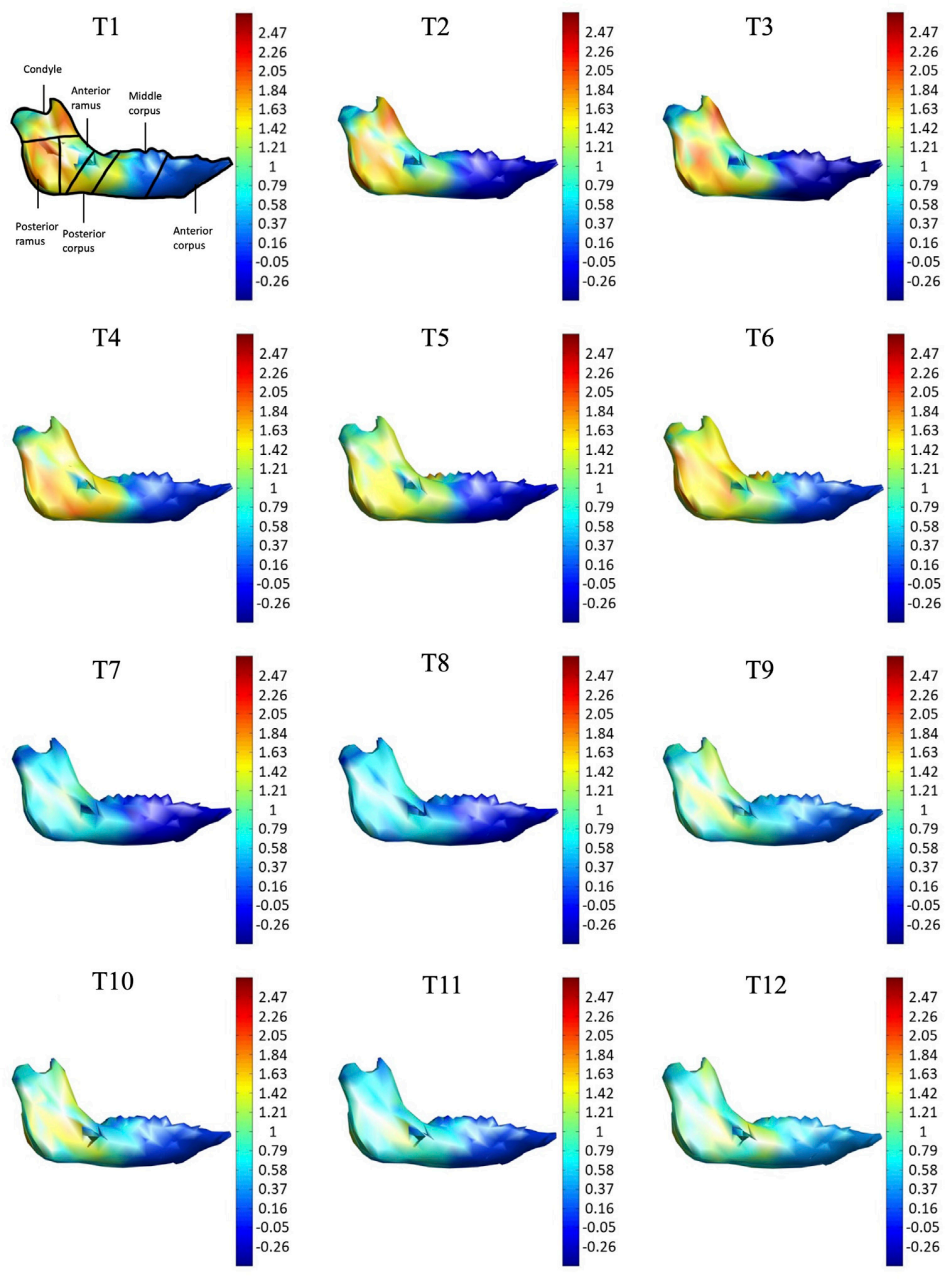
Lateral view of the mandible of a typical subject with monthly rates of morphological changes over the mandibular surface as color maps from T1 to T12. The models were scaled to a similar size for better visualization. (unit: %/month).

As shown in the lateral and superior views, the mandible appeared to change its size over the 12-month growing period but with largely similar shapes (Figures 2, 3). The bone thickness of the anterior and posterior ramus regions and the mediolateral width of the condylar head also increased over time (Figure 4). As seen from the lateral view, the growth rate of the mandible showed an anteriorly decreasing trend from the posterior part of the ramus to the anterior corpus edge (Figure 5). The condyle region showed the fastest growth rate during the initial growth period (T1-T4), with an average growth rate of about 1.19%/month, 1.03%/month, and 0.85%/month in the anterior/posterior, superior/inferior, and medial/lateral direction respectively (Table 4). The second-fastest growth rate occurred in the posterior ramus with average growth rates of about 0.75, 0.62, and 0.53%/month in the anterior/posterior, superior/inferior, and medial/lateral directions, respectively, over T1-T12 (Table 4). The slowest growth rates occurred in the middle corpus region with average values of about 0.39, 0.39, and 0.32%/month in the anterior/posterior, superior/inferior, and medial/lateral directions, respectively; and in the anterior corpus region with corresponding values of 0.53, 0.45, and 0.37%/month (Table 4). In the anterior/posterior direction, the fastest growth of the condyle occurred at T1-T2, while the most rapid growth of the other regions occurred at T2-T3 (Table 4). In the superior/inferior direction, the fastest growth of the condyle and ramus regions occurred at T1-T2, while the most rapid growth of the corpus regions occurred at T2-T3 (Table 4).

**TABLE 4:** Means and standard deviations (SD) of the monthly averaged anterior/posterior (A/P), superior/inferior (S/I), medial/lateral (M/L), and total growth rates over each of the six regions of the mandible over the monitoring period of one year. (*n* = 8; unit: %/month).

		T1-T2	T2-T3	T3-T4	T4-T5	T5-T6	T6-T7	T7-T8	T8-T9	T9-T10	T10-T11	T11-T12	Ensemble	
													Mean	SD
Condyle	A/P	1.92	1.53	0.72	0.61	0.95	0.58	0.61	0.61	0.54	0.47	0.42	0.81	0.48
		(0.05)	(0.06)	(0.03)	(0.03)	(0.04)	(0.03)	(0.04)	(0.04)	(0.02)	(0.03)	(0.03)		
	S/I	1.52	1.37	0.72	0.54	0.77	0.46	0.39	0.48	0.49	0.37	0.48	0.69	0.39
		(0.03)	(0.03)	(0.02)	(0.02)	(0.03)	(0.03)	(0.02)	(0.02)	(0.03)	(0.03)	(0.03)		
	M/L	1.04	1.23	0.63	0.49	0.73	0.40	0.41	0.38	0.49	0.30	0.31	0.58	0.30
		(0.07)	(0.03)	(0.02)	(0.02)	(0.03)	(0.02)	(0.02)	(0.02)	(0.02)	(0.01)	(0.02)		
Posterior Ramus	A/P	1.55	1.58	0.76	0.64	0.74	0.57	0.63	0.53	0.46	0.42	0.39	0.75	0.42
		(0.04)	(0.05)	(0.03)	(0.02)	(0.03)	(0.03)	(0.04)	(0.03)	(0.03)	(0.03)	(0.02)		
	S/I	1.41	1.19	0.64	0.45	0.58	0.42	0.43	0.38	0.46	0.41	0.43	0.62	0.35
		(0.03)	(0.03)	(0.02)	(0.02)	(0.03)	(0.02)	(0.02)	(0.02)	(0.03)	(0.02)	(0.02)		
	M/L	1.16	0.95	0.51	0.47	0.60	0.44	0.32	0.36	0.39	0.30	0.32	0.53	0.28
		(0.03)	(0.02)	(0.02)	(0.02)	(0.03)	(0.02)	(0.02)	(0.02)	(0.02)	(0.01)	(0.02)		
Anterior Ramus	A/P	1.43	1.50	0.70	0.60	0.77	0.58	0.58	0.54	0.46	0.43	0.42	0.73	0.38
		(0.05)	(0.07)	(0.03)	(0.03)	(0.04)	(0.03)	(0.03)	(0.04)	(0.03)	(0.02)	(0.02)		
	S/I	1.36	1.21	0.65	0.48	0.66	0.47	0.46	0.36	0.43	0.38	0.50	0.63	0.34
		(0.03)	(0.03)	(0.02)	(0.01)	(0.02)	(0.02)	(0.03)	(0.02)	(0.02)	(0.02)	(0.03)		
	M/L	1.06	1.12	0.41	0.42	0.63	0.42	0.30	0.30	0.40	0.31	0.31	0.52	0.30
		(0.03)	(0.03)	(0.02)	(0.01)	(0.03)	(0.02)	(0.02)	(0.01)	(0.02)	(0.02)	(0.01)		
Posterior corpus	A/P	0.54	0.88	0.65	0.61	0.81	0.44	0.75	0.50	0.50	0.42	0.49	0.60	0.16
		(0.04)	(0.06)	(0.05)	(0.05)	(0.05)	(0.03)	(0.07)	(0.04)	(0.06)	(0.03)	(0.03)		
	S/I	0.45	0.55	0.54	0.59	0.77	0.40	0.50	0.45	0.59	0.47	0.50	0.53	0.10
		(0.03)	(0.03)	(0.05)	(0.05)	(0.05)	(0.03)	(0.04)	(0.04)	(0.07)	(0.04)	(0.04)		
	M/L	0.36	0.53	0.42	0.54	0.62	0.45	0.58	0.50	0.51	0.40	0.36	0.48	0.09
		(0.03)	(0.03)	(0.03)	(0.04)	(0.05)	(0.03)	(0.08)	(0.05)	(0.04)	(0.03)	(0.03)		
Middle corpus	A/P	0.49	0.68	0.32	0.33	0.35	0.42	0.34	0.29	0.34	0.27	0.42	0.39	0.12
		(0.02)	(0.04)	(0.02)	(0.02)	(0.02)	(0.03)	(0.02)	(0.01)	(0.02)	(0.02)	(0.03)		
	S/I	0.56	0.60	0.32	0.41	0.40	0.42	0.33	0.30	0.29	0.29	0.37	0.39	0.11
		(0.04)	(0.03)	(0.02)	(0.03)	(0.02)	(0.03)	(0.02)	(0.02)	(0.02)	(0.02)	(0.02)		
	M/L	0.37	0.45	0.25	0.33	0.32	0.41	0.27	0.30	0.27	0.23	0.29	0.32	0.07
		(0.02)	(0.02)	(0.02)	(0.02)	(0.02)	(0.04)	(0.02)	(0.02)	(0.02)	(0.01)	(0.01)		
Anterior corpus	A/P	0.69	0.89	0.48	0.42	0.58	0.50	0.56	0.47	0.43	0.42	0.36	0.53	0.15
		(0.04)	(0.06)	(0.03)	(0.03)	(0.03)	(0.04)	(0.06)	(0.03)	(0.03)	(0.03)	(0.02)		
	S/I	0.57	0.71	0.43	0.33	0.46	0.40	0.41	0.37	0.43	0.36	0.44	0.45	0.11
		(0.03)	(0.04)	(0.03)	(0.02)	(0.03)	(0.03)	(0.03)	(0.02)	(0.03)	(0.03)	(0.03)		
	M/L	0.42	0.54	0.32	0.31	0.41	0.35	0.36	0.32	0.36	0.32	0.36	0.37	0.07
		(0.03)	(0.03)	(0.02)	(0.02)	(0.04)	(0.02)	(0.04)	(0.03)	(0.02)	(0.02)	(0.02)		

**FIGURE 6:**
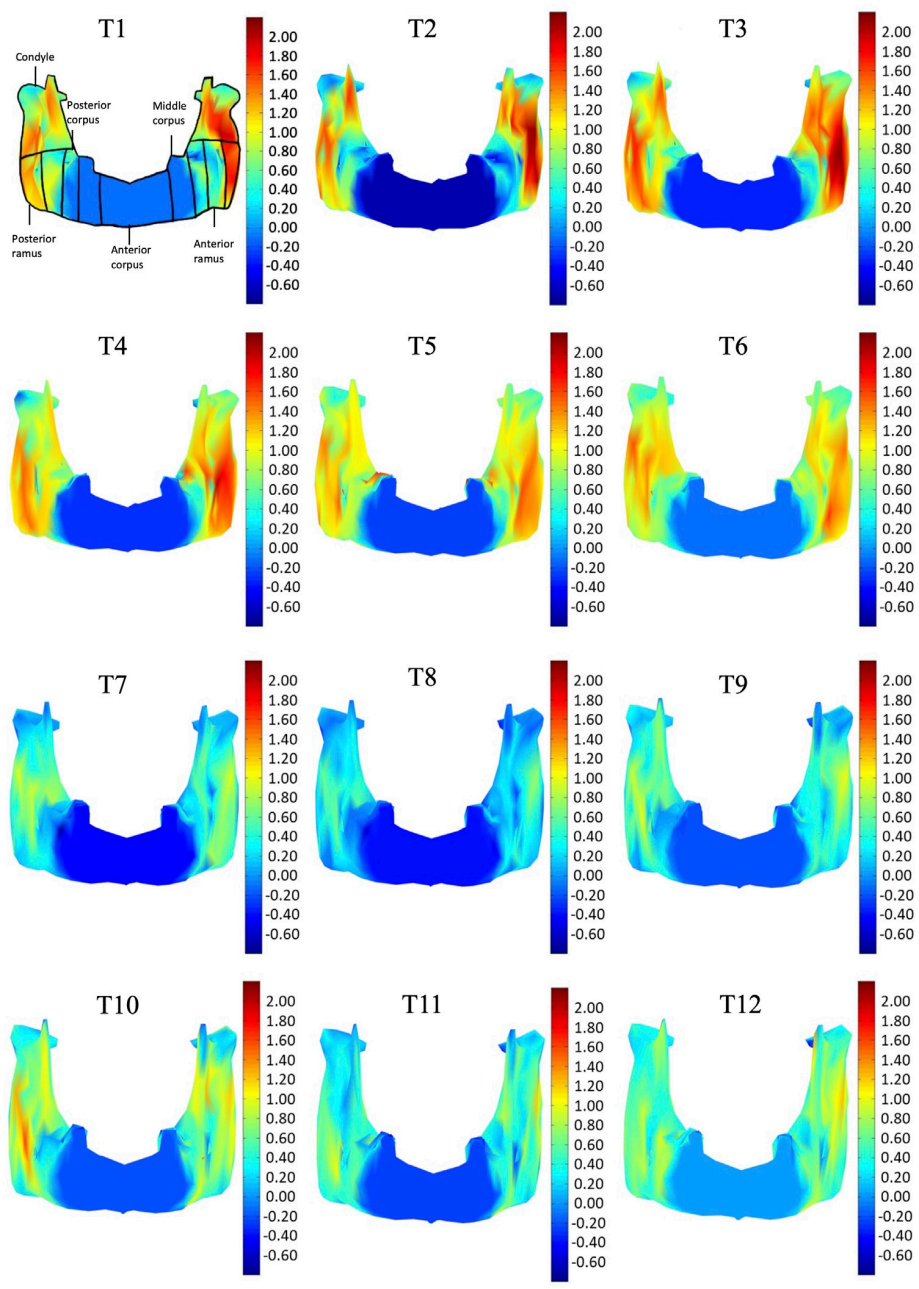
Frontal view of the mandible of a typical subject with monthly rates of morphological changes over the mandibular surface as color maps from T1 to T12. The models were scaled to a similar size for better visualization. (unit: %/month).

**FIGURE 7:**
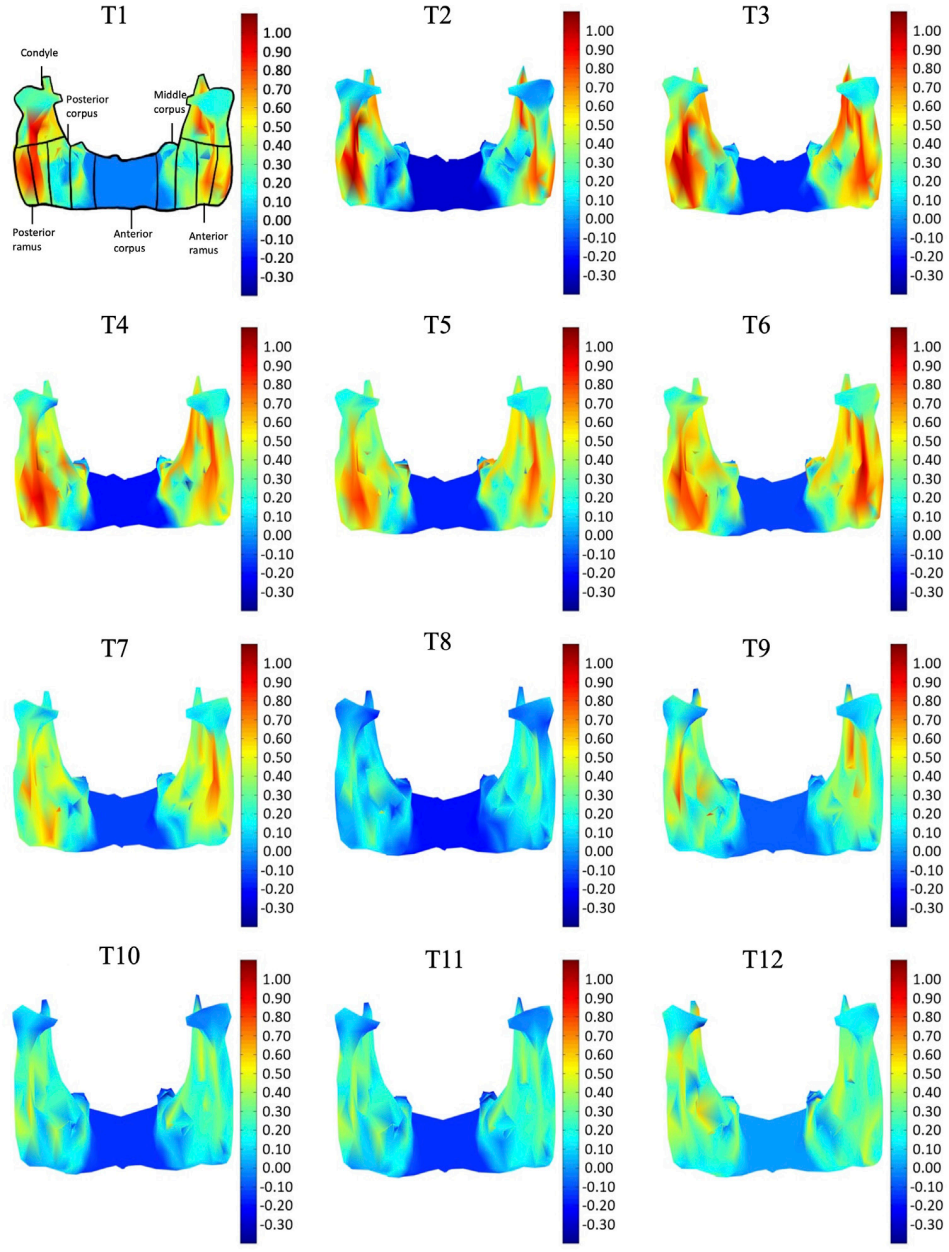
Posterior view of the mandible of a typical subject with monthly rates of morphological changes over the mandibular surface as color maps from T1 to T12. The models were scaled to a similar size for better visualization. (unit: %/month).

**FIGURE 8:**
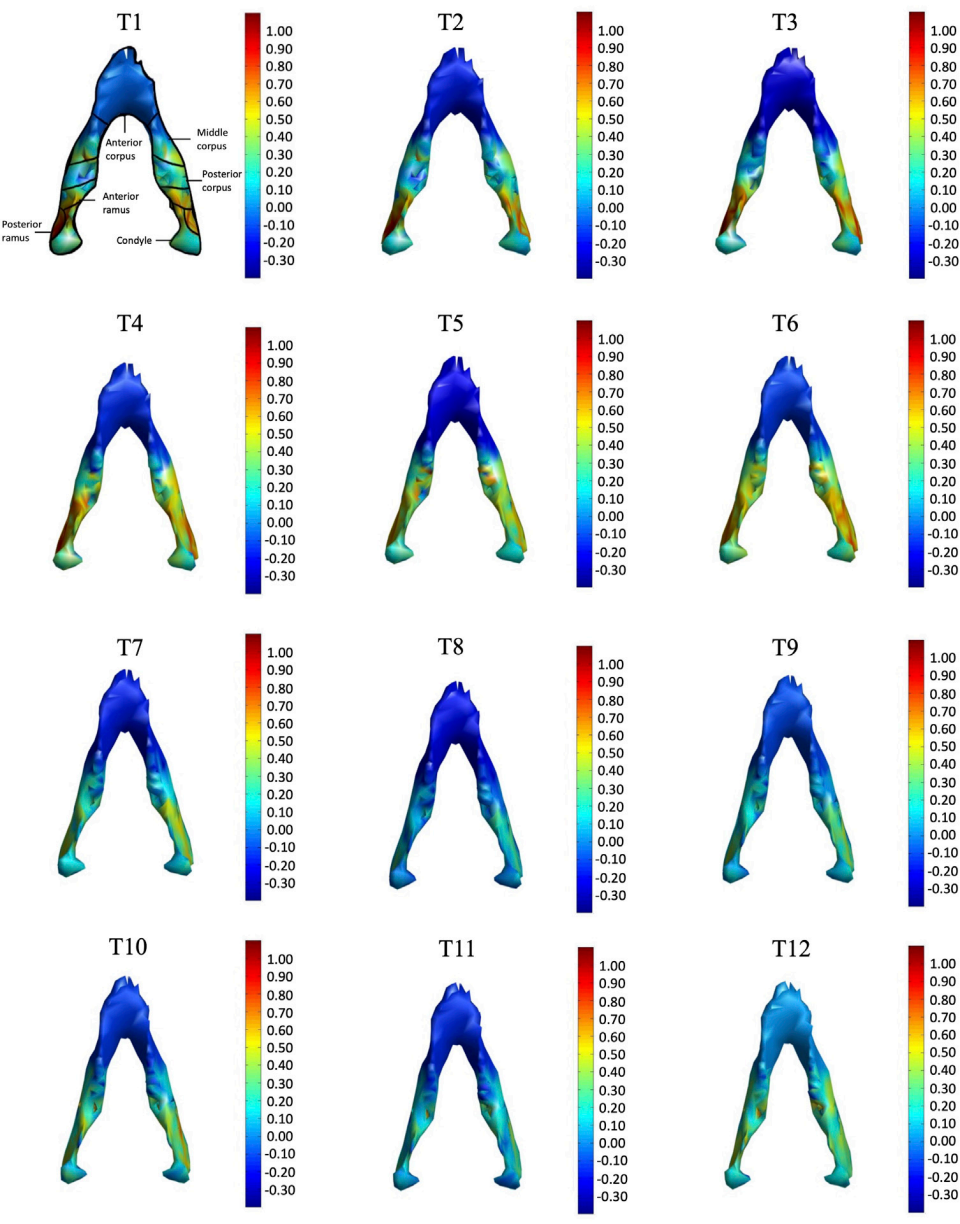
Superior view of the mandible of a typical subject with monthly rates of morphological changes over the mandibular surface as color maps from T1 to T12. The models were scaled to a similar size for better visualization. (unit: %/month).

**FIGURE 9:**
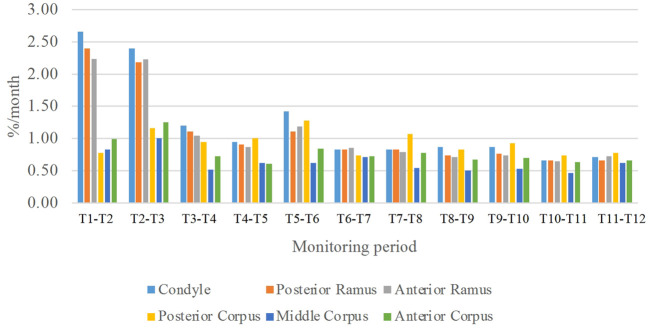
Means of the monthly average growth rates over each of the six regions of the mandible over the monitoring period of one year. (*n* = 8)

In the frontal and posterior views, the largest growth rate (a total rate of about 2.66%/month) also occurred in the condyle, both medial and lateral sides (Figures 5–8). The second-fastest growth rate (a total rate of about 2.40%/month) occurred in the posterior ramus, while the lowest growth rates were in the middle and anterior corpus regions, with total rates of about 0.46 and 0.64%/month, respectively (Figures 5–8 and Table 4). The condyle had a similar growth rate as the anterior and posterior ramus regions from T1 to T12 (Figure 9 and Table 4). In the medial/lateral direction, the fastest growth of the posterior ramus occurred at T1-T2, and the fastest growth of the condyle, anterior ramus, middle, and anterior corpus occurred at T2-T3 (Table 4). The most rapid growth of all the regions occurred in the anterior/posterior direction, except the middle corpus (Table 4). The results of the superior view further confirmed the patterns seen in the other views (Figure 8).

## Discussion

The current study aimed to develop a new approach based on 3D time-dependent, non-linear osteometric modeling of the bones to describe the 3D quantitative, continuous patterns of the morphological changes of the mandible during growth from the CBCT data of miniature pigs during the growth over the first 12 months. The continuous growth patterns of the mandible during the 12-month monitoring period were obtained, averaged over six selected anatomical parts, and displayed as continuous color maps over the surface of the mandible. Generally, the growth rate of the mandible was found to decrease anteriorly, with the condyle region and the posterior part of the ramus growing faster than the rest of the mandible. The condyle region showed the fastest growth rate and the posterior ramus the second during the growth period, while the middle and anterior corpus regions showed the slowest growth rates. The current results suggest that the new approach will be useful for future studies on the growth of the mandible in different animals.

The new approach developed in the current study enabled the monitoring and description of the 3D mandibular growth for each vertex on the surface and within the body of the mandible at high accuracy over a long period of time. This is in contrast to previous studies that described the growth of the mandible in terms of the changes in the distances between two landmarks (Lin H.-S. et al., 2014). Line segments defined by two landmarks only revealed the gross patterns of the growth. More detailed information for a small area that cannot be defined by two landmarks could not be obtained. For example, the condyle or coronoid process could not be measured with two apparent landmarks. Another limitation of previous approaches is that the descriptions were mostly in two dimensions. A planar description cannot reveal the growth changes of the overall mandible because it is three-dimensional in shape and nature.

The CBCT-based measurement method and the new analysis technique based on 3D osteometric modeling used in the current study enabled the quantification of the 3D growth in terms of the monthly changes of the vertex points on and within the mandible. This new approach involved between-model registration and time-fitting to simulate the non-linear growth of the mandible. The method searched for the corresponding points on the bone models at two consecutive instances in time with the highest match between anatomical and morphological features. The RMSE of the positions between the new method and the gold standard for the positions of the 17 anatomical landmarks over the 12 months of monitoring were small (less than 0.86 mm), especially over the first eight months of monitoring (less than 0.44 mm). These validation results indicate that the new approach developed in the current study could be used for measuring the quantitative, continuous patterns of growth of the mandibular bone. While quantitative values of the average growth rates were reported over the six selective parts of the mandible, continuous growth maps over the mandibular bone surface were also helpful for a general picture of the growth patterns. Color maps could not be generated for the growth rates for points within bones; further development of display methods for these points will be needed for the ease of clinical applications.

With the current three-dimensional modeling approach, the overall mandible was described by hundreds of triangular elements, the rates of morphological changes of which were indicated by color-codes. This was in contrast to previous representations of mandible growth using length changes between landmarks. For example, landmarks Go and GT have been used to represent the length of the mandible, and its distance changes used to describe mandibular growth. It is noted that bony growth between Go and GT was assumed to have a linear trend. However, the current results showed that the bony growth rates were non-linear between Go and GT, decreasing gradually from Go to GT (Figure 5). Similar to Go-GT, growth rates along the line joining landmarks Cd-lat and Go, indicating the mandible height, were also found to be non-linear, increasing from the lateral pole of the condylar head (Cd-lat) to the ramus body (Go) (Figure 6). These results suggest that the inter-marker distances may affect the representation of bony growth using line segments. The current new approach to finding correspondence between any two models over time enabled us to describe the growth and growth rates (or morphological changes) for each vertex on the bone model. Since the vertices were very close to each other in terms of the accuracy of the CBCT, the growth rates could be described in much greater detail than traditional line segment representations.

The growth patterns of the current pigs’ mandibles during the first four months appeared to be related to the germination of the initial mandibular dentition, which begins from the third lower deciduous incisors and the deciduous canines immediately after birth, followed by the first deciduous incisors (Cheng et al., 2010). To provide space for the germination of the anterior dentition, the anterior corpus region in four-week-old pigs appeared to have grown to a size suitable for the growth of the incisors and canines, as shown in the first CT scan and the reducing rate of changes in sizes for the rest of the monitoring period. Subsequent to the first CT scan, the condyle and posterior ramus regions were found to have the fastest growth rate during T1-T4, with the largest monthly change in the condyle part (Table 3, 4 and Figures 5–8). The mandible became taller and longer as a result of the continuous growth of the condyle and the middle and posterior corpus regions, which was accompanied by germination and growth of the posterior teeth (Figure 5). The current results suggest that the size of the condylar region at the time of the first CT scan and the subsequent morphological changes of the mandible in the early period of growth are helpful for the development of the temporomandibular joint for mandibular movement and the germination of the mandibular dentition.

The current study was limited to quantifying the surface morphological changes and the average rates of such changes over six selected anatomical parts of the mandible during the 12-month monitoring period, showing the non-uniform change patterns of different regions of the mandible to describe the overall growth. The proposed approach can be further extended to include volumetric information of the mandible to provide further information on the morphological changes at any point within the mandible bone during growth. It is noted that without implanting markers within the bone, it was challenging to indicate the locations and amount of bone apposition and resorption that resulted in the morphological changes observed in the current study and to identify any bone rotation within the mandible. On the other hand, the mandibular growth was monitored over 48 weeks until the pig became too big to be imaged by the current CBCT machine, so the growth patterns reported were limited to the period from eruption up to 2/3 of the mixed dentition stage. In the future, advances in imaging techniques may reduce harmful ionizing radiation to such low levels that the proposed approach could safely be validated in human subjects.

## Conclusions

The current study presented for the first time in the literature the 3D continuous morphological changes of the mandible in miniature pigs during the growth of the first 12 months as average morphological changes over six selected anatomical parts and continuous changes over the surface of the mandible using color maps. This was achieved by integrating CBCT and a new osteometric modeling approach, which quantified the non-linear patterns and non-linear rate of morphological changes in different growth regions and the whole mandible. The current approach will be useful for future studies on the growth of the mandible in other animals and the exploration of the growth of different dentition patterns, such as protruded or retruded mandibles, which may contribute to a complete understanding of the underlying growth factor of dental occlusion.

## Data Availability

The original contributions presented in the study are included in the article/Supplementary Material, further inquiries can be directed to the corresponding authors.
